# Determining the Research Priorities for Adult Primary Brain Tumours in Australia and New Zealand: A Delphi Study with Consumers, Health Professionals, and Researchers

**DOI:** 10.3390/curroncol29120781

**Published:** 2022-12-17

**Authors:** Georgia K. B. Halkett, Lauren J. Breen, Melissa Berg, Rebecca Sampson, Hao-Wen Sim, Hui K. Gan, Benjamin Y. Kong, Anna K. Nowak, Bryan W. Day, Rosemary Harrup, Melissa James, Frank Saran, Brett Mcfarlane, Chris Tse, Eng-Siew Koh

**Affiliations:** 1 Curtin School of Nursing/Curtin Health Innovation Research Institute, Faculty of Health Sciences, Curtin University, Bentley, WA 6102, Australia; 2 Curtin School of Population Health/Curtin enAble Institute, Faculty of Health Sciences, Curtin University, Bentley, WA 6102, Australia; 3 School of Nursing and Midwifery, Faculty of Medicine, Nursing, Midwifery and Health Sciences, The University of Notre Dame Australia, Fremantle, WA 6160, Australia; 4 NHMRC Clinical Trials Centre, University of Sydney, Camperdown, NSW 2050, Australia; 5 School of Clinical Medicine, Faculty of Medicine and Health, University of New South Wales, Sydney, NSW 2052, Australia; 6 Department of Medical Oncology, Chris O’Brien Lifehouse, Camperdown, NSW 2050, Australia; 7 Department of Medical Oncology, The Kinghorn Cancer Centre, Darlinghurst, NSW 2010, Australia; 8 Cancer Therapies and Biology Group, Centre of Research Excellence in Brain Tumours, Olivia Newton-John Cancer Research Institute, Austin Hospital, Heidelberg, VIC 3084, Australia; 9 School of Cancer Medicine, La Trobe University, Bundoora, VIC 3086, Australia; 10 Department of Medicine, University of Melbourne, Carlton, VIC 3010, Australia; 11 Department of Medical Oncology, Royal North Shore Hospital, St Leonards, NSW 2065, Australia; 12 Medical School, University of Western Australia, Nedlands, WA 6009, Australia; 13 Department of Medical Oncology, Sir Charles Gairdner Hospital, Nedlands, WA 6009, Australia; 14 QIMR Berghofer Medical Research Institute, Brisbane, QLD 4006, Australia; 15 Royal Hobart Hospital, Hobart, TAS 7000, Australia; 16 Canterbury Regional Cancer and Haematology Service, Christchurch 8011, New Zealand; 17 Department of Medicine, University of Otago, Christchurch 8011, New Zealand; 18 Department of Blood and Cancer, Auckland City Hospital, Auckland 1023, New Zealand; 19 Cooperative Trials Group for Neuro-Oncology (COGNO), Camperdown, NSW 2050, Australia; 20 Brain Tumour Support NZ, Hamilton 3210, New Zealand; 21 International Brain Tumour Alliance, London W1B 2AD, UK; 22 South West Sydney Clinical School, UNSW Medicine, University of New South Wales, Liverpool, NSW 2170, Australia; 23 Liverpool and Macarthur Cancer Therapy Centres, Liverpool, NSW 2170, Australia; 24 Ingham Institute for Applied Medical Research, Liverpool, NSW 2170, Australia

**Keywords:** research priorities, brain tumours, delphi, consumers, research barriers, research enablers

## Abstract

The aim of this project was to determine research priorities, barriers, and enablers for adult primary brain tumour research in Australia and New Zealand. Consumers, health professionals, and researchers were invited to participate in a two-phase modified Delphi study. Phase 1 comprised an initial online survey (*n* = 91) and then focus groups (*n* = 29) which identified 60 key research topics, 26 barriers, and 32 enablers. Phase 2 comprised two online surveys to (1) reduce the list to 37 research priorities which achieved consensus (>75% 2-point agreement) and had high mean importance ratings (*n* = 116 participants) and (2) determine the most important priorities, barriers, and enablers (*n* = 90 participants). The top ten ranked research priorities for the overall sample and sub-groups (consumers, health professionals, and researchers) were identified. Priorities focused on: tumour biology, pre-clinical research, clinical and translational research, and supportive care. Variations were seen between sub-groups. The top ten barriers to conducting brain tumour research related to funding and resources, accessibility and awareness of research, collaboration, and process. The top ten research enablers were funding and resources, collaboration, and workforce. The broad list of research priorities identified by this Delphi study, together with how consumers, health professionals, and researchers prioritised items differently, and provides an evidence-based research agenda for brain tumour research that is needed across a wide range of areas.

## 1. Introduction

The term adult primary brain tumours includes both malignant and benign tumours. The Australian Institute of Health and Welfare estimated that 1879 new cases of adult brain tumours would be diagnosed and 1518 people would die from brain cancer in Australia in 2020 [1]. Currently, there is only a 22% chance of surviving at least five years from diagnosis of malignant brain cancer, a much lower survival rate than many other cancers [1,2,3]. Although clinical trials of different treatment regimens have been conducted, mean survival for high-grade malignant brain tumours (WHO grade 4) continues to be short at 12–15 months [4]. The only substantive clinical trial to result in a survival difference was published in 2005 [5,6]. There is an unmet need for research to improve survival and quality of life particularly for these patients with extremely poor survival and a potentially large tumour-related symptom burden. However, research to improve treatment and management of patients with benign tumours is also needed.

Although brain tumours are classified as rare cancers, there are currently a limited number of research grant opportunities available in neuro-oncology through the Brain Cancer Mission and Australian Medical Research Future Funding and other funding opportunities. Nevertheless, funding and resources for brain tumour research remain limited compared to that available for other tumour types [7,8]. Therefore, it is important to understand current research priorities for research focusing on adult brain tumours.

In 2010 Cancer Council NSW conducted a three-stage Delphi Study (*n* = 18 health professional participants and 2 consumers) to determine research priorities for adult gliomas in Australia in 2010 [9]. The top 10 research priorities were: (1) a national glioma collaborative network (clinical data, DNA, tumour specimens, protocols and infrastructure); (2) molecular identification of glioma subsets and druggable targets; (3) molecular and pharmacogenetic determinants of treatment response; (4) tumour biology, molecular determinants of tumour behaviour and understanding cellular heterogeneity of glioma; (5) mechanisms of glioma migration and invasion; (6) clinical trials structures to fast-track therapeutic combinations, using novel biological or imaging endpoints; (7) imaging which identifies robust features for (a) earlier detection of response and b) differentiates progression from pseudo-progression; (8) supportive care needs of patients, families and carers, and appropriate services and education; (9) techniques to detect drug resistant gliomas earlier in order to increase effectiveness of chemotherapy; and (10) evaluate the role of the nurse coordinator in patient care from diagnosis to the end of life. It is now over a decade since this the 2010 Delphi study was conducted and the landscape is changing. In Australia advocacy has resulted in the formation of the Australian Brain Cancer Mission, which aims to facilitate national research, clinical, and funding efforts [10]. A key objective identified is to ensure patients have the opportunity to participate in clinical trials. Several centres of excellence for neuro-oncology have also been established. Additionally, the Cooperative Trials Group for Neuro-Oncology (COGNO) continues to conduct a range of multidisciplinary, large scale national investigator-initiated clinical trials. Therefore, COGNO believed it was time to conduct this Delphi study to determine the current priorities for brain tumour research. The aim of this study was to determine the priorities for adult primary brain tumour research in Australia and New Zealand. A secondary aim was to determine barriers and enablers for conducting research in this area.

While the focus of our study was on Australian and New Zealand research priorities and barriers and enablers, this study should contribute to the global discussion about research priorities for adult brain tumours and help to advance research in this field. Additionally, this research will inform funding agencies in Australasia about current priorities.

## 2. Materials and Methods

### 2.1. Study Design

**Figure 1:**
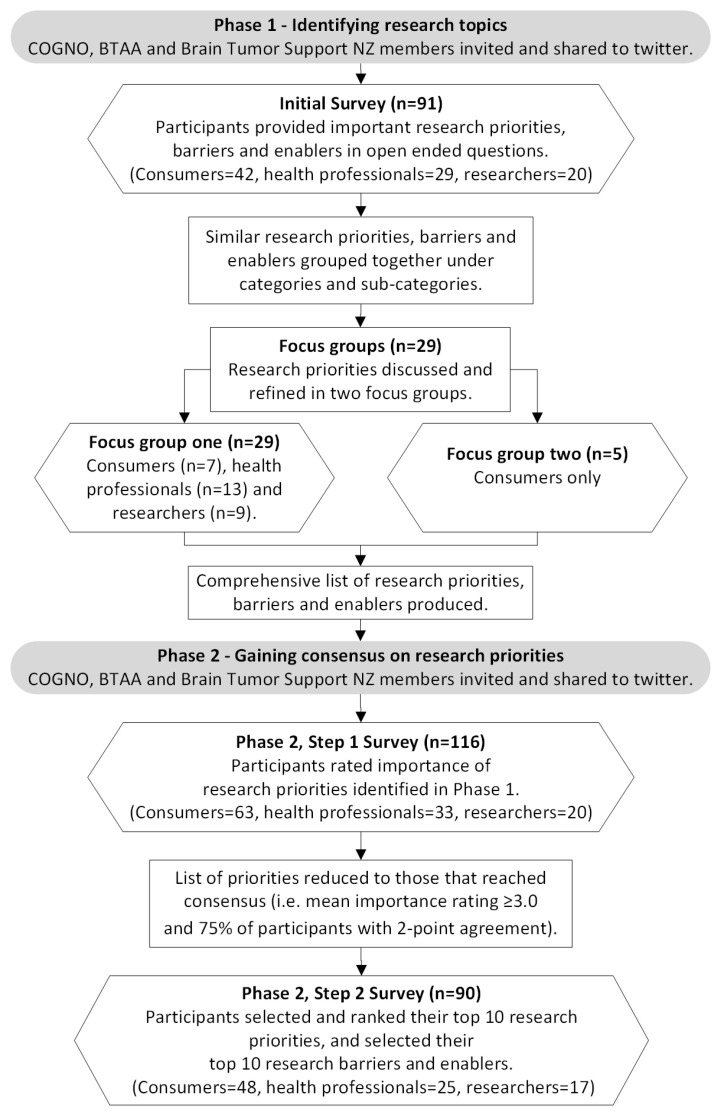
Overview of the research process. Brain Tumour Alliance Australia (BTAA), Cooperative Trials Group for Neuro-Oncology (COGNO), New Zealand (NZ).

A mixed-methods design was used with a modified Delphi methodology. A Delphi study involves a group of experts in an iterative process consisting of several rounds of enquiry, with subsequent rounds informed by a summary of previous rounds, for the purpose of “the formation of consensus or the exploration of a field beyond existing knowledge and the current conceptual world” [11]. While most Delphi studies have 10 to 100 experts, sample sizes vary considerably and no set sample size is advocated because it is dependent on the topic, its complexity, required relevant perspectives, and the range of expertise required [12]. Our sample included participants with diverse perspectives and experiences of brain tumours so we aimed for a larger sample size of 100 for the survey components of the study. The study was conducted in two Phases. Phase 1 focused on identifying research topics and Phase 2 comprised two survey rounds focused on gaining consensus on research priorities (see Figure 1). The method used to conduct the study was informed by a recent Delphi study conducted by Butow et al. [13] and previous Delphi studies by members of the authorship team (GH and LB) [14,15,16,17]. The reporting standards for the Conducting and REporting of DElphi Studies (CREDES) [11] were used to guide the conduct of the study. Ethics approval for all stages of the study was gained from Curtin University Human Research Ethics Committee (HRE2021-0317).

### 2.2. Phase 1, Step 1 Survey

#### 2.2.1. Participants

An email was sent out to the multidisciplinary membership of COGNO (*n* = 833) [18] and consumer groups in Australia and New Zealand (Brain Tumour Alliance Australia [BTAA] and Brain Tumour Support New Zealand) inviting members to complete a survey eliciting research priorities. The email provided a link to the information sheet, consent form, and questionnaire (Qualtrics, Provo, UT, USA). Social media (Twitter and LinkedIn) and word of mouth were also used to recruit participants. Health professionals, researchers, and consumers were eligible to participate. Consumer participation was integral to our Delphi study as their involvement is likely to lead to the conduct of projects that are prioritised by the community (rather than investigator-driven projects) and improve outcomes for people diagnosed with brain tumours overall [19,20]. Consumers are actively involved in COGNO and all clinical trials run through COGNO have involvement from the COGNO consumer advisory panel.

#### 2.2.2. Survey Instrument

The Step 1 survey was open to participants from 17 June 2021 to 23 July 2021. Open-ended questions asked participants to identify up to five research priorities, barriers to research, enablers for research, and the five priorities Australia and New Zealand are best positioned to address in relation to primary adult brain tumours [13]. Participants also completed demographic questions (including gender, location, country/state, age, and their primary role in relation to adult brain tumours (consumer, health professional or researcher).

### 2.3. Phase 1, Step 2 Focus Groups

Two virtual focus groups were held via Microsoft Teams. Each focus group was run by two experienced facilitators (GH, LB) and a research assistant (RS). Focus group participants from the health professional and researcher groups were invited if they opted into participating in focus groups in the initial survey and were selected based on craft groups and state. Eight consumers (four patients and four carers) from the list of consumers who opted into focus groups were also invited to participate. During the focus groups, the research priorities identified in Step 1 were discussed and refined into a comprehensive list of priorities for the research team to work with.

At the request of consumers, a second focus group was held and consumers who attended the first focus group were invited to allow them to freely voice their opinions in greater detail and provide feedback on the wording of the research priorities.

### 2.4. Phase 2 Delphi Process

An online Delphi process with two rounds of feedback was conducted in Phase 2.

#### 2.4.1. Participants

To be eligible to participate, individuals had to identify as part of one of three groups; those that work as health care professionals with adults with primary brain tumours, as researchers, or as consumers, which included patients, carers, and advocates. Participants from Phase 1 were invited to Phase 2, Step 1, and additional invitations were sent out via COGNO’s (*n* = 862) [21], BTAA’s, and Brain Tumour Support New Zealand’s mailing lists and social media. Participants who completed the Phase 2, Step 1 survey were eligible to participate in the Phase 2, Step 2 survey.

#### 2.4.2. Phase 2, Step 1

The Phase 2, Step 1 survey was open to participants from 21 September 2021 to 19 October 2021. The survey (Supplementary Materials File S1) focused on research priorities and asked participants to rate each of the 60 research priorities identified in phase one, on a four-point scale from 1 being “not important; not a priority” to 4 being “very important; urgent priority”. Alternatively, participants could select ‘unable to score’ or could skip questions.

Research priorities were presented in the order that had previously been used in the focus groups. Category headings were not shown to avoid prejudicing participants’ opinions of the individual research priorities that were identified.

The consensus of a research priority was defined as >75% of participants rating the research priority within two points, where the priority mean rating was ≥3.0. There is no clear guideline in the literature for a consensus cut-off, with previous studies having used proportions of agreement from 51% to 80% [22]. We adapted the approach of previous Delphi studies [13,22] which used a 5-point scale to our study which used a 4-point scale. Data were summarised and used to formulate questions to be included in Phase 2, Step 2.

#### 2.4.3. Phase 2, Step 2

Eligible participants were contacted directly via email with a link to the Phase 2, Step 2 survey. The Phase 2, Step 2 survey was open to participants from 11 November 2021 to 6 December 2021. Participants were sent up to two follow-up reminder emails if they had not started or finished their survey. Thirty-eight research priorities from the Phase 2, Step 1 survey reached the consensus criterion and were carried forward to the Phase 2, Step 2 survey. The order of the list of research priorities was based on mean importance ratings from Phase 2, Step 1 (highest to lowest). Participants were asked for their opinion of the top 10 research priorities and were then asked to rank their 10 selected priorities from highest to lowest priority. Participants were also asked to identify up to five of their ten selected priorities that they believed COGNO could lead, or be closely involved in, and advocate for their importance (COGNO-specific data will be reported elsewhere).

This Step 2 survey also included a list of 26 barriers and 32 enablers to research in primary adult brain tumours that were identified by participants and refined by the research team in Phase 1. The order of the list of barriers and enablers was randomised for each participant. Participants were asked to select what they believed were the top 10 research barriers and enablers from these lists. Finally, participants were asked to select the top 5 of their 10 selected research enablers which they believed COGNO could lobby or advocate for to reduce barriers to conducting research in adult primary brain tumours (COGNO-specific data will be reported elsewhere).

### 2.5. Data Analysis

#### 2.5.1. Phase 1

Survey responses were analysed using content analysis [23]. Responses that were similar were grouped together under categories and sub-categories. Counts of repeated items were also kept. Content analysis was reviewed by at least three members of the team at each stage.

Focus group categories and sub-categories were reviewed continuously by the team for repetition of similar priorities, wording, and order of presentation of the items. During these reviews, it was decided to remove the categories and sub-categories for presentation in future surveys so that participants focused on the priorities rather than the categories and sub-categories.

#### 2.5.2. Phase 2

Statistical analysis was completed using IBM SPSS Version 27. Incomplete surveys were included in the analysis, with missing data for each section identified. Descriptive statistics i.e., frequencies, percentages, means, and standard deviations (SD), are presented in tables. Chi-square tests or Kruskal-Wallis 1-way ANOVAs were used to explore differences between participant groups in demographic characteristics at each round. Fisher-Freeman-Halton Exact Tests (FETs) are reported when Chi-square cross-tabulations had >20% cells with low expected counts (<5). In Phase 2, Step 1, importance ratings were collapsed within 2 points (i.e., 1 and 2, 2 and 3, 3 and 4) to determine priorities with >75% agreement.

In Phase 2, Step 2, rankings of research priorities were reverse weighted (i.e., 1st = 10 and 10th = 1) and means were adjusted by setting zero values if a participant did not select the priority. To adjust for group sizes in the mean rank of the overall group, sub-group means and standard deviations were averaged. Kruskal-Wallis one-way ANOVAs were used to explore differences between participating groups in rankings. To adjust for different sub-group sizes, when percentage proportions of the total cohort are presented, counts were multiplied by a factor that adjusted the sub-group size to be one third of the total group. A *p* value < 0.05 was deemed to be statistically significant.

## 3. Results Phase 1

### 3.1. Phase 1, Step 1 Survey

Of 246 participants who started the Phase 1, Step 1 survey, 91 participants (42 consumers, 29 health professionals, and 20 researchers) (37%) provided a response for at least one research priority and were used for analysis (Table 1). Indirect distribution prevented response rates from being calculated. Cross tabulations of groups by gender, location, and country were not significantly different nor was there a significant effect on the group by age.

Consumer participants included the following: previously diagnosed = 21, carer = 16, advocate = 2, missing = 1 (total = 40). Health professional participants included the following disciplines: medical oncologist = 10, neurosurgeon = 5, radiation oncologist = 3, nurse = 1, cancer care coordinator = 1, neuro-oncologist = 1, clinical trial nurse = 1, nuclear medicine physician = 1, neuropathologist = 1, unknown = 1 (total = 25). It was noted that many of the participants who identified primarily as health professionals were also actively involved in conducting brain tumour research. Researchers included the following specialty areas: psychosocial research = 9, pre-clinical research = 5, clinical trials = 3, imaging research = 1, drug research = 1, unknown = 1 (Total = 20).

A total of 370 initial research priorities were identified. The research team removed duplicates and grouped the priorities into categories and sub-categories. The final list of 176 priorities was summarised into six categories with 26 sub-categories. The categories were: epidemiology; tumour biology (e.g., biobanking, registries, diagnostics); clinical and translational research (clinical trials, primary care/early diagnosis, surgical, systemic treatment [chemotherapy, immunotherapy], radiation therapy, imaging, low-grade glioma, treatment outcomes, symptom management); supportive care (quality of life, models of care, palliative care, clinical practice guidelines/optimal care pathways, brain injury, cognition, and specific support); psychosocial support (psycho-oncology, survivorship, carer support, financial toxicity, patient education/communication, complementary and alternative therapies), and research infrastructure priorities (funding). The research infrastructure priorities which focussed on funding were incorporated into the analysis of barriers and enablers.

In addition, 337 barriers and 274 enablers were identified by participants in this survey. The barriers and enablers identified in Step 1 were discussed within the research team. This list was refined and reduced to 26 barriers and 32 enablers.

### 3.2. Phase 1, Step 2 Focus Groups

In the first focus group, participants included health professionals (*n* = 13), researchers (*n* = 9), and consumers (*n* = 7), total = 29 (demographics—Table 2). Focus group participants were shown the categories/sub-categories and list of priorities and invited to provide feedback. This process enabled us to further reduce the list of research priorities to 145. However, this list contained overlapping items that required further refinement. During the second focus group, five of the consumers discussed the research priorities presented and provided suggestions on appropriate wording which would facilitate consumer participation in Phase 2.

Further team meetings with the investigator group (manuscript authors), discussions, emails, and a working document with edit tracking were used to further refine this list to 60 research priorities. The final lists of research priorities, barriers, and enablers formed the quantitative surveys used in Phase 2.

## 4. Results Phase Two: Consensus on Research Priorities

### 4.1. Participants

Of 151 participants who started the Phase 2, Step 1 survey, 116 provided responses that were used for analysis. Indirect distribution prevented response rates from being calculated. Consumer participants included the following: previously diagnosed = 33, carer = 23, advocate = 7 (total = 63). Health professional participants included the following disciplines: medical oncologist = 14, neurosurgeon = 8, radiation oncologist = 6, nurse = 1, cancer care coordinator = 1, neuro-oncologist = 1, neuropathologist = 1, radiographer = 1, (total = 33). Similar to Phase 1, many participants who identified as health professionals are also actively involved in conducting brain tumour research. Researchers included the following speciality areas: psychosocial research = 9, pre-clinical research = 7, clinical trials = 2, imaging research = 1, and drug research = 1 (total = 20). Other participant details are summarised in Table 3.

There was a significantly greater proportion of female consumers (75%) than female health professionals (42%, X^2^ [2, *n* = 116] = 10.115, *p* = 0.006). There was a significant effect of group on age overall (H [2] = 10.120, *p* = 0.006); consumers were significantly older (Median = 55.0 years) compared with health professionals (Median = 49.0, *p* = 0.042) and researchers (Median = 44.5 years, *p* = 0.025). No other pairwise comparisons were significant. Cross tabulations of role by location and country were not significantly different.

Of 116 eligible participants, 100 began the Step 2 survey and 90 (48 consumers, 25 health professionals, and 17 researchers) provided responses (response rate = 78%) that were used for the analysis (as participants could skip questions, there was a maximum of 89 responses for any question). All health professionals in this step were clinicians (neurosurgeons, radiation oncologists, medical oncologists, neuropathologists) actively involved in clinical trials. See Table 3 for participant demographics. There was a significantly greater proportion of female consumers (78%) compared with female health professionals (48%, X^2^ [2, *n* = 90] = 9.062, *p* = 0.012). Cross tabulations of role by location and country were not significantly different. There was a significant effect of group/role on age overall (H [2] = 7.958, *p* = 0.019); consumers were significantly older (Median = 54.5 years) compared with researchers (Median = 46.0 years, *p* = 0.030). No other pairwise comparisons were significant.

### 4.2. Step 1 Survey—Research Priority Importance Ratings

Table 4 summarises the mean importance ratings and percent agreement for items included in the Step 1 survey. There were no significant differences between groups in the proportion of consumers, health professionals, and researchers who dropped out before the end of the survey (FET, *p* = 0.577).

After Step 1, 23 items were removed because they met the criteria for deletion (<75% agreement and mean <3.0).

### 4.3. Step 2 Survey—Ranking Research Priorities, Enablers, and Barriers

#### 4.3.1. Sensitivity Analysis

There were no significant differences between groups in the total number of options selected for each of the following questions: priorities (*p* = 1.000), barriers (*p* = 0.628) or enablers selected (*p* = 0.059), or priorities ranked (*p* = 1.000) (Max. possible = 10); or priorities (*p* = 0.294) or enablers (*p* = 0.538) opted for COGNO (Max. possible = 5). More consumers selected ‘unable to comment’ for the question about selected research priorities for COGNO (consumers = 5, health professionals = 1, and researchers = 0).

A sensitivity analysis was performed on the type of participants who missed questions. Gender, location, or role were not significant predictors for missing the questions for selecting priorities (FET, *p* = 0.289, *p* = 1.000, *p* = 0.467 respectively), barriers (FET, *p* = 0.299, *p* = 1.000, *p* = 1.000 respectively) or enablers (FET, *p* = 0.212, *p* = 1.000, *p* = 0.293 respectively), or ranking priorities (FET, *p* = 0.072, *p* = 0.104, *p* = 0.810 respectively); or enablers (FET, *p* = 0.195, *p* = 0.474, *p* = 0.422 respectively) for COGNO. Gender or role were not significant predictors for missing opting priorities for COGNO (FET, *p* = 0.467, *p* = 0.130 respectively). However, a significantly greater proportion of regional/rural (29%) or remote (50%) participants missed opting for priorities for COGNO compared with participants located in a major city (7%, FET, *p* = 0.028).

Age did not significantly predict missing the questions for selecting priorities (U = 59.5, z = 0.578, *p* = 0.667) or enablers (U = 142.0, z = 0.373, *p* = 0.734), or opting priorities (U = 488.0, z = 1.456, *p* = 0.145) or enablers (U = 194.00, z = 1.750, *p* = 0.084) for COGNO. Participants who missed ranking priorities were significantly older (Median = 62.5) compared with participants who answered (Median = 51.0) (U = 280.0, z = 2.381, *p* = 0.013).

#### 4.3.2. Research Priorities and Rankings

Table 5 presents participants’ selection of the top ten research priorities and rankings. It also demonstrates rankings that were significantly different between groups. The figure in Supplementary Materials Figure S1 contains a visual representation of each subgroup’s ranking of the overall top fifteen priorities. The line graph figure in Supplementary Materials Figure S2 demonstrates the change in overall and sub-group mean rankings of the 1st to 37th ranked priority in Table 5.

#### 4.3.3. Barriers

**Figure 2:**
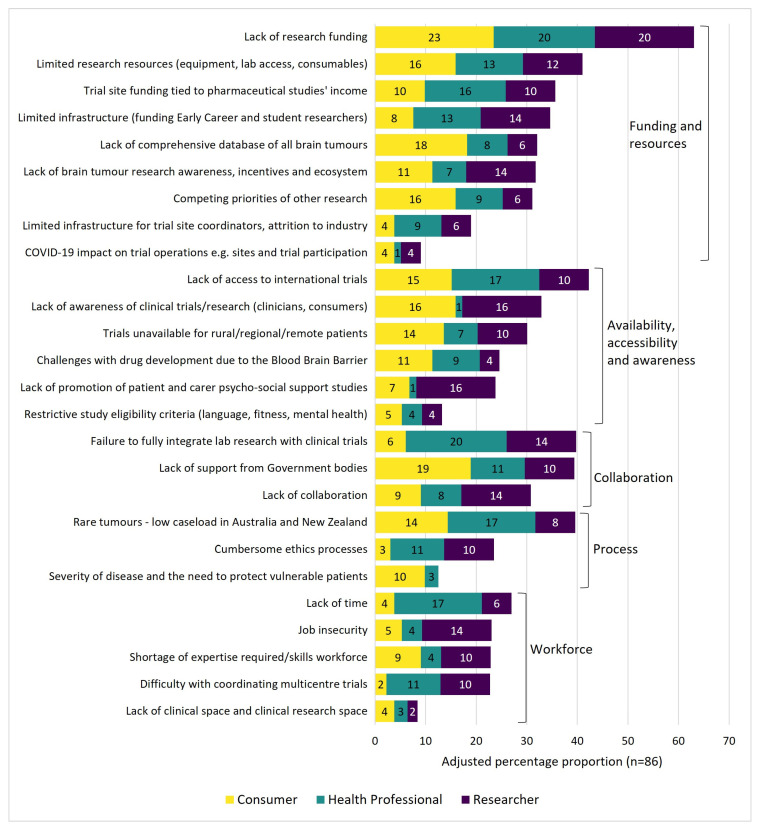
The adjusted proportion of participants by sub-group who selected research barriers in the top ten.

Figure 2 summarises the barriers to conducting brain tumour research from the highest to the lowest proportion of participants. The top ten barriers to conducting brain tumour research were in the categories of funding and resources (*n* = 5), availability, accessibility and awareness (*n* = 2), collaboration (*n* = 2), and process (*n* = 1). The top five barriers for the overall group were: lack of research funding; limited research resources; lack of access to international trials; failure to fully integrate lab research with clinical trials; and limited research opportunities for rare tumours with a low caseload in Australia and New Zealand.

Lack of research funding was the most frequently selected barrier within each sub-group and for the overall group. Four of the consumer groups’ and three of the researcher groups’ equal top five barriers were in the funding and resources category. The workforce-related barriers of lack of time and job insecurity were within the top five barriers for the health professional and researcher groups respectively.

#### 4.3.4. Enablers

**Figure 3:**
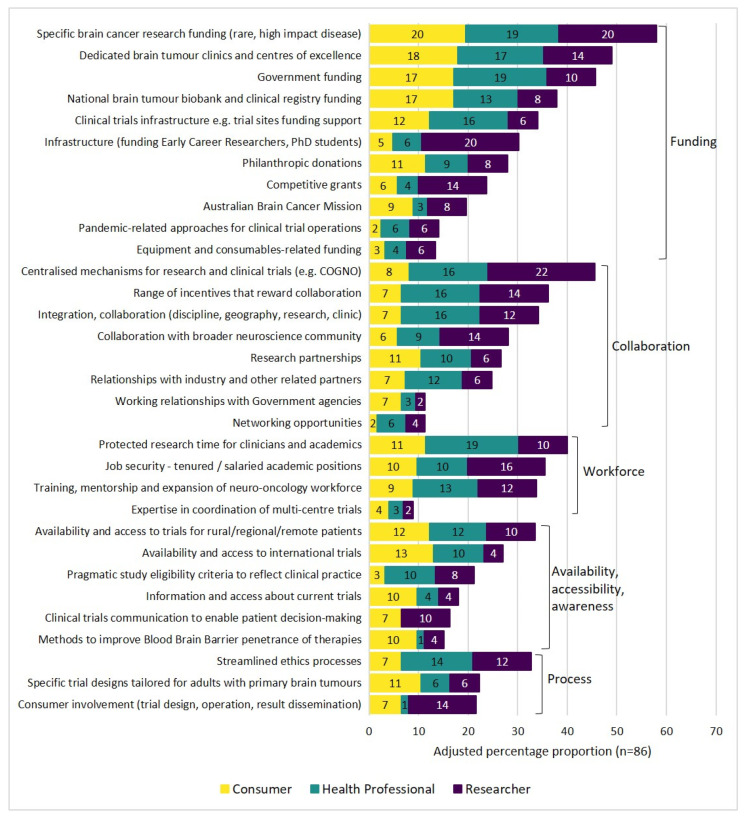
The adjusted proportion of participants by sub-group who selected research enablers in the top ten.

Figure 3 summarises participants’ top ten selected enablers ordered from the highest to the lowest proportion of participants. The top ten enablers to conducting brain tumour research were in the categories of funding and resources (*n* = 5), collaboration (*n* = 3), and workforce (*n* = 2). The first four enablers for the overall group, and the health professional and consumer sub-groups, were in the funding and resources category. The equal top five research enablers for the overall group were specific brain cancer research funding (rare, high-impact disease); dedicated brain tumour clinics and centres of excellence; government funding; national brain tumour biobank and clinical registry funding; protected research time for clinicians and academics; and centralised mechanisms for research and clinical trials (e.g., COGNO).

The two enablers (1) specific funding allocated to brain cancer research, in recognition of the additional challenges to conducting research in a rare (but high impact) disease, and (2) dedicated brain tumour clinics and centres of excellence were in the top five selected enablers within each sub-group and for the overall group.

## 5. Discussion

This Delphi study determined the current top research priorities for adult brain tumours in Australia and Zealand. The overall top five ranked research priorities in the current study were: conducting clinical trials that include relevant patient-reported outcomes; understanding the tumour microenvironment with the aim of reducing immunosuppression and facilitating immunotherapy; exploring the effectiveness of precision medicine/personalised treatment based on genomic profiling; conducting pre-clinical research to identify actionable drivers (and new molecular targets) for brain tumour therapy, and investigating advanced neuro-oncology imaging for diagnosis and treatment response monitoring.

A strength of this study is that we have included opinions from many participants, including a large cohort of health consumers. For a niche area, such as rare primary brain tumours, we had strong representation across all major clinical disciplines, researchers, and consumers. There was a variation between consumers, health professionals, and researchers’ rankings of priorities, including two priorities in the top 10 that were significantly different, which became most apparent in priorities ranked 11th to 37th by the overall group. The following priorities were in the top 10 for all three groups: understanding the tumour microenvironment with the aim of reducing immunosuppression, and facilitating immunotherapy, exploring the effectiveness of precision medicine/personalised treatment based on genomic profiling, and investigating reasons for treatment resistance.

Conducting clinical trials that include relevant patient-reported outcomes (including quality of life or unmet supportive care needs) was ranked highest overall, ranked second by consumers, and first by researchers. Interestingly, this was not identified in the top 10 priorities by health professionals (ranked 16th). All health professionals who participated in this final step of the study were clinicians actively involved in clinical trials. Health professionals may have put this item as a lower priority because they focus on treatment outcomes in their day-to-day practice and the underlying evidence-based generated from research which has outcomes using non-patient reported data such as survival, toxicities, and recurrence. There has been a recent drive to include patient-reported outcomes in clinical trial grant applications, and also in clinical practice as a measure of health-care quality [24]; COGNO has access to psychosocial researchers and services such as Cancer Quality of Life Service Team (CQUEST) at University of Technology Sydney who provide advice on including patient-reported outcomes in trials. A recent Delphi study by Mazariego et al. [25] determined priority recommendations for the implementation of patient-reported outcomes in clinical cancer care which could guide the inclusion of patient-reported outcomes in future brain tumour research and clinical care.

Immunotherapy is a current medical research priority in many solid cancers with two topics in the top 10 priorities focusing on this. The last decade has seen exponential growth in immunotherapy treatments and research which likely explains the emergence of this topic in this Delphi study. Brown et al. [26] recently described the use of immunotherapy in brain tumours and Chuntova et al. [27] described the challenges of using immunotherapy for glioblastoma. A review of the types of immunotherapies to treat glioblastoma outlined glioblastoma vaccines, oncolytic viral therapies, immune-checkpoint inhibitors, and chimeric antigen T cell therapy [28]. Additionally, Khasraw et al. [29] investigated the role of PD-1 inhibitors and described their potential role in the treatment of glioblastoma. Currently, clinical trials are being designed to include combination immunotherapy for patients with glioblastoma to provide efficacy data and demonstrate meaningful survival benefits [30].

A top priority identified in the current study was to explore the effectiveness of precision medicine/personalised treatment based on genomic profiling. This builds on the previous priority identified in 2010 as determining the molecular and pharmacogenetic determinants of treatment response to enable the development of tailored treatment regimens that offer improved quality of life and survival [9]. A recent systematic review evaluated current molecular and pre-clinical studies, concluding that there is a need for further experimental research in this area [31]. Additionally, Panovska and Smet [32] highlight that precision oncology initiatives might improve outcomes for glioblastoma patients.

The research priority of conducting pre-clinical research to identify actionable drivers (and new molecular targets) for brain tumour therapy builds on the previous Delphi study in 2010 [9] which reported “better identification of glioma subsets by molecular characteristics and identification of druggable targets” as a top 10 priority. In the current study, this was the health professionals’ top-ranked priority, which they ranked significantly higher than both consumers and researchers. This may reflect a growth in understanding of the value of molecular profiling in prognostication and guiding therapy in the last decade and the identification of druggable target mutations and driver mutations [33,34]. To understand molecular evolution, there is a need to have supporting infrastructure in order to collect the samples and perform comprehensive molecular profiling, such as that being done by the large-scale collaboration of the Glioma Longitudinal Analysis (GLASS) consortium [35]. In Australia, the LUMOS (Low and Intermediate Grade Glioma Umbrella Study of Molecular Guided TherapieS) trial is currently being conducted [36] and the Stafford Fox Rare Cancer Research program and Omico/MoST studies will expand the understanding of rare or progressing CNS tumours [37,38].

Analogous to the findings from the 2010 Delphi study [9], which highlighted the need for novel imaging research as a priority area, there continues to be a priority to investigate advanced neuro-oncology imaging for diagnosis and treatment response monitoring. A 2015 neuro-oncology priority setting partnership in the UK between patients, carers, and health professionals determined the top 10 clinical uncertainties of interventions for brain and spinal cord tumours, which were framed as research questions and included a question related to scanning for tumour recurrence [39]. Following priority setting for brain tumour research in the UK, two Cochrane Reviews were conducted on the use of imaging: one on scanning intervals [40] and the other on Magnetic Resonance perfusion for diagnostics [41]. Other studies of interest focusing on imaging in brain tumours include studies on using volumetric measurements of mutant IDH inhibition in non-enhancing diffuse gliomas in Phase 1 studies [42] and using fluoroethyltyrosine (FET) positron emission tomography (PET) to assist in radiation therapy planning of glioblastoma [43]. A subsequent multicentre trial is now being conducted in Australia to evaluate (FET-PET) imaging in radiotherapy planning and clinical management of people with glioblastoma (the FIG study) (ACTRN12619001735145).

In 2010, the highest-ranked research priority was the formation of a national glioma collaborative network (including clinical data) [9]. The current study also highlighted the importance of establishing and maintaining a brain tumour registry as a top 10 priority. This work is progressing in Australia with Matsuyama et al. [44] recently publishing work on the selection of clinical quality indicators for an Australian Brain Cancer Registry. Another new priority identified in the current study was developing national benchmarking and quality indicators and outcomes of care (including surgery) to improve the quality and efficacy of treatment.

Health professionals ranked priorities in the areas of clinical and translational research studies significantly higher compared with other groups, including phase 0 studies and using liquid biopsies for diagnosis and monitoring. From the consumer perspective, there is not yet widespread awareness of these clinical trial and translational research topics as they are not mainstream and would not routinely be discussed with their oncologists. Recently, Sanai [45] provided strategies for conducting Phase 0 clinical trials. Research is also being conducted in the areas of proteomics [46] and using serum microRNA as a biomarker for post-operative monitoring in glioma [47].

Researchers ranked developing psychosocial interventions for unmet needs and evaluation of optimal models of care coordination in their top 5 priorities and significantly higher than either health professionals or consumers (note that 47% of researchers produced psycho-oncology research). Both these priorities build on the 2010 Delphi study [9] which highlighted both the development of appropriate support and education materials and the evaluation of the contribution of nurse coordinators in the role of providing care for patients. Our team has conducted studies to determine the unmet needs of patients diagnosed with high-grade glioma [48] and their carers [49,50] and also developed an intervention to assist in carer preparedness following diagnosis [51,52]. Additionally, work is being conducted in Australia to understand current support services available to patients [53,54] and to develop and test psychosocial interventions that provide additional support to patients [55]. Internationally several studies have been conducted on patient and carer needs and interventions to support them [56,57,58,59]. Additionally, recent funding has been allocated in Australia through the Australian Brain Cancer Mission to continue this work and improve supportive care for patients diagnosed with brain cancer and their carers. This work continues to be a priority in psycho-oncology with more studies required to support patients and carers following a diagnosis.

Consumers ranked the following research priorities significantly higher than health professionals and/or researchers: understanding the causes of adult brain tumour development and conducting drug repurposing studies in adult brain tumours. When diagnosed with a brain tumour or when caring for someone with a brain tumour, patients and carers may be trying to process and construct meaning around the cause of their diagnosis to help them cope and adjust to their recent diagnosis [60]. Additionally, with such a poor prognosis they may also be searching for other treatments leading to better outcomes, which also leaves them open to false or misleading remedies [61].

Other top 10 research priorities identified in the current study that were not included in the 2010 study include investigating reasons for treatment resistance and developing pre-clinical models and strategies to enhance blood-brain barrier penetration for novel drugs. Several studies have collected or are collecting molecular information to enable further understanding of gliomas and treatments [35,36]. The importance of the blood-brain barrier was highlighted by Sarkaria et al. [62] who assessed whether glioblastomas disrupt the blood-brain barrier using clinical data.

Additionally, this study identified the common barriers and enablers for adult brain tumour research. Five of the top ten research barriers and enablers are related to the funding and resources category, highlighting that this is an important area of need for brain tumour research. Funding and resources-related barriers included that trial site funding was tied to pharmaceutical studies’ income or there was a lack of or limited research funding, research resources, infrastructure (funding early career and student researchers), and a comprehensive database of all brain tumours. These findings build on a previous survey conducted with Australian medical students which identified that barriers to incorporating research into their medical careers included low funding, job insecurity, and low salaries [63]. Enablers to address the issues of limited funding and resources identified in the current study included specific brain tumour research funding, in consideration of its rarity but as high impact disease, government funding, dedicated brain tumour clinics and centres of excellence, the national brain tumour biobank and clinical registry funding, and clinical trials infrastructure e.g., trial sites funding support. Currently, there are research grant opportunities available for research in neuro-oncology through competitive funding initiatives such as the Australian Brain Cancer Mission and the Australian government’s Medical Research Future Fund. Top research barriers in other categories were availability, accessibility, and awareness, as well as collaboration and process (rare tumours with a low caseload in Australia and New Zealand). Other top research enablers were related to collaboration and the workforce. Studies have shown that workplace features such as feeling valued and supported, having access to appropriate training, and quality communication are relevant to workforce sustainability [64].

### 5.1. Recommendations

The key findings of this Delphi study will be integral to informing the strategic plan for COGNO’s research priority focus areas over the coming 5–10 years. The research priorities highlight the areas of opportunities for potential breakthrough developments for primary brain tumours, which should aim to ultimately improve the patient and carer experience, and include novel targeted and immune therapeutics; advanced imaging and liquid biopsy technologies, improved understanding of pharmacokinetics/pharmacodynamics, tumour microenvironment and resistance mechanisms, and successful translation of research findings into national practice. Leveraging a strategic plan, there is potential for researchers to make meaningful contributions to the international research effort in these domains. Before proceeding, it will be important for COGNO to establish the research currently underway, both in Australia and internationally, which has synergy with these domains, to determine and focus on areas where Australia and/or New Zealand will have a competitive advantage, and to establish pragmatic approaches to attenuate the effect of research barriers. It is acknowledged that the differences in consumers, health professionals and researchers’ rankings of priorities need to be considered in creating and communicating a plan that ensures the efficient use of scarce resources. Arguably, the feasibility and tractability of the research priority should also be considered. Some of the priorities, such as benchmarking and quality measures, are service developments which could be best supported through public funding. The study findings also provide evidence with which to advocate to the various funding bodies in Australia and New Zealand and organisations working in the brain tumour space.

### 5.2. Limitations

This study was limited to Australian and New Zealand participants. The response rate in this study, when it was possible to calculate, was comparable to rates reported in another Australian Delphi study involving a similar participant group [65]. However, our sample may not be representative of all clinicians, researchers, and consumers in this field. We also note that we only had small numbers of participants in some professional groups (e.g., nuclear medicine physician = 1, neuropathologist = 1); however, this is representative of these specialty groups in neuro-oncology. The research priorities presented to participants were on broad topics and not all participants were able to decide on all items. The lists of research priorities presented in Phase 2, Step 1 survey may have been biased because they were presented in a particular order. Instead, items could have been randomised. However, we kept the logical order from the focus groups which grouped similar topic areas near each other to aid participants in completing the survey.

## 6. Conclusions

This study provides a summary of research priorities for adult brain tumours from the perspectives of consumers, health professionals, and researchers. The overall top five ranked research priorities focused on conducting clinical trials that include relevant patient reported outcomes, understanding the tumour microenvironment to facilitate immunotherapy, exploring the effectiveness of precision medicine/personalised treatment based on genomic profiling, conducting pre-clinical research to identify actionable drivers for brain tumour therapy, and investigating advanced neuro-oncology imaging for diagnosis and treatment response monitoring. The broad list of research priorities identified by this Delphi study, together with how consumers, health professionals, and researchers prioritised items differently, demonstrates that the brain tumour research portfolio is broad and research is needed in a wide range of areas. Funding and resources were highlighted as an important area of need with many research barriers and enablers in these categories. Designing research projects which seek to incorporate and address these priorities will lead to improved outcomes for patients diagnosed with adult brain tumours. We have also identified barriers and enablers to conducting research which will assist future planning in this area.

## Previous presentations

Findings from a part of this study were presented at the 14th COGNO Annual Scientific Meeting (2022) as an oral presentation.

## Figures and Tables

**Table 1:** Phase 1, Step 1 participant demographics for survey.

Characteristic	Consumers ^a^ *n* = 40 (100%)	Health Professionals ^b^ *n* = 25 (100%)	Researchers *n* = 20 (100%)	Total *n* = 85 (100%)
**Gender** Male Female				
	11 (27.5)	13 (52.0)	7 (35.0)	31 (36.5)
	29 (72.5)	12 (48.0)	13 (65.0)	54 (63.5)
**Location Type** Major City Regional/Rural Remote				
	33 (82.5)	22 (88.0)	20 (100.0)	75 (88.2)
	7 (17.5)	3 (12.0)	0 (0)	10 (11.8)
	0 (0)	0 (0)	0 (0)	0 (0)
**Location** Australia *New South Wales* *Victoria* *Queensland* *Western Australia* *Australian Capital Territory* *Tasmania* *South Australia* New Zealand				
	35 (87.5)	22 (88.8)	19 (95.0)	76 (89.4)
	14 (40.0)	10 (45.5)	7 (36.8)	31 (40.8)
	8 (22.9)	8 (36.4)	2 (10.5)	18 (23.7)
	4 (11.4)	3 (13.6)	3 (15.8)	10 (13.2)
	2 (5.7)	0 (0)	6 (31.6)	8 (10.5)
	2 (5.7)	0 (0)	0 (0)	2 (2.6)
	3 (8.6)	1 (4.5)	0 (0)	4 (5.3)
	2 (5.7)	0 (0)	1 (5.3)	3 (3.9)
	5 (12.5)	3 (12.0)	1 (5.0)	9 (10.6)
**Age ^c^**	**Mean (SD)**	**Mean (SD)**	**Mean (SD)**	**Mean (SD)**
	50.8 (10.8)	49.3 (9.0)	46.3 (11.0)	49.3 (10.4)

^a^ Two consumers did not provide demographic information. ^b^ Four health professionals did not provide demographic information. ^c^ One health professional did not provide their age. Note: Location type was self-reported and not defined.

**Table 2:** Phase 1, Step 2 demographics for focus groups.

Characteristic	Consumers *n* = 7 (100%)	Health Professionals *n* = 13 (100%)	Researchers *n* = 9 (100%)	Total *n* = 29 (100%)
**Gender** Male Female				
	4 (57.1)	7 (53.8)	4 (44.4)	15 (51.7)
	3 (42.9)	6 (46.2)	5 (55.6)	14 (48.3)
**Location Type** Major City Regional/Rural Remote				
	7 (100.0)	11 (84.6)	9 (100.0)	27 (93.1)
	0 (0)	2 (15.4)	0 (0)	2 (6.9)
	0 (0)	0 (0)	0 (0)	0 (0)
**Location** Australia *New South Wales* *Victoria* *Queensland* *Western Australia* *Australian Capital Territory* *Tasmania* *South Australia* New Zealand				
	5 (71.4)	11 (84.6)	9 (100.0)	25 (86.2)
	2 (40.0)	4 (36.4)	2 (22.2)	8 (32.0)
	1 (20.0)	6 (54.5)	2 (22.2)	9 (36.0)
	0 (0.0)	0 (0.0)	2 (22.2)	2 (8.0)
	1 (20.0)	0 (0.0)	3 (33.3)	4 (16.0)
	1 (20.0)	0 (0.0)	0 (0.0)	1 (4.0)
	0 (0.0)	1 (9.1)	0 (0.0)	1 (4.0)
	0 (0.0)	0 (0.0)	0 (0.0)	0 (0.0)
	2 (28.6)	2 (15.4)	0 (0.0)	4 (13.8)
**Age**	**Mean (SD)**	**Mean (SD)**	**Mean (SD)**	**Mean (SD)**
	52.9 (7.5)	52.8 (9.6)	46.9 (8.8)	50.9 (9.0)

**Table 3:** Participant type, personal characteristics, and location of Phase 2 (Step 1 and Step 2) participants.

Characteristic	Step 1				Step 2
	Consumers *n* = 63 (100%)	Health Professionals *n* = 33 (100%)	Researchers *n* = 20 (100%)	Total *n* = 116 (100%)	Total *n* = 90 (100%)
**Gender** Male Female					
	16 (25.4)	19 (57.6)	6 (30.0)	41 (35.3)	26 (28.9)
	47 (74.6)	14 (42.4)	14 (70.0)	75 (64.7)	64 (71.1)
**Location Type** Major City Regional/Rural Remote					
	45 (71.4)	31 (93.9)	18 (90.0)	94 (81.0)	74 (82.2)
	15 (23.8)	2 (6.1)	2 (10.0)	19 (16.4)	14 (15.6)
	3 (4.8)	0 (0.0)	0 (0.0)	3 (2.6)	2 (2.2)
**Location** Australia *New South Wales* *Victoria* *Queensland* *Western Australia* *Australian Capital Territory* *Tasmania* *South Australia* New Zealand					
	61 (96.8)	29 (87.9)	20 (100.0)	110 (94.8)	85 (94.4)
	23 (37.7)	9 (31.0)	9 (45.0)	41 (37.3)	33 (38.8)
	15 (24.6)	11 (37.9)	3 (15.0)	29 (26.4)	23 (27.1)
	8 (13.1)	4 (13.8)	3 (15.0)	15 (13.6)	9 (10.6)
	4 (6.6)	1 (3.4)	5 (25.0)	10 (9.1)	8 (9.4)
	6 (9.8)	2 (6.9)	0 (0.0)	8 (7.3)	6 (7.1)
	2 (3.3)	2 (6.9)	0 (0.0)	4 (3.6)	3 (3.5)
	3 (4.9)	0 (0.0)	0 (0.0)	3 (2.7)	3 (3.5)
	2 (3.2)	4 (12.1)	0 (0.0)	6 (5.2)	5 (5.6)
**Age**	**Mean (SD)**	**Mean (SD)**	**Mean (SD)**	**Mean (SD)**	**Mean (SD)**
	53.9 (13.7)	48.0 (10.2)	45.7 (11.3)	50.8 (12.8)	51.3 (11.9)

**Table 4:** Phase 2, Step 1 participants importance ratings and consensus on research priorities.

Refer-ence Number	Research Priority	Consumers *n* = 63		Health Professionals *n* = 33		Researchers *n* = 20		Overall *n* = 116			
								Mean (SD)	Agreement (2 Point) *n* (%)	Topic Retained	*n*
		Mean (SD)	*n*	Mean (SD)	*n*	Mean (SD)	*n*				
1	Understanding the causes of adult brain tumour development	3.5 (0.74)	63	3.0 (0.77)	33	3.5 (0.69)	20	3.4 (0.76)	98 (84.5)	Yes	116
2	Developing research questions around familial glioma syndromes	3.1 (0.87)	61	2.2 (0.68)	33	2.8 (0.62)	18	2.8 (0.87)	80 (71.4)	No	112
3	Pre-clinical research to identify actionable drivers (and new molecular targets) for therapy	3.6 (0.58)	62	3.5 (0.71)	33	3.2 (0.71)	19	3.5 (0.65)	108 (94.7)	Yes	114
4	Improved pre-clinical models of gliomas and other primary Central Nervous System malignancies	3.5 (0.65)	61	3.4 (0.74)	33	3.3 (0.84)	18	3.4 (0.71)	104 (92.9)	Yes	112
5	Understanding the tumour microenvironment and immunosuppression to facilitate immunotherapy	3.7 (0.53)	61	3.5 (0.75)	33	3.5 (0.62)	17	3.6 (0.62)	105 (94.6)	Yes	111
6	Pre-clinical models and strategies to enhance Blood Brain Barrier (BBB) penetration for novel drugs	3.6 (0.60)	62	3.1 (0.74)	33	3.3 (0.59)	18	3.4 (0.68)	101 (89.4)	Yes	113
7	Novel therapeutic approaches with the ability to specifically target the stem cell-like population	3.5 (0.68)	59	3.2 (0.82)	33	3.2 (0.79)	18	3.4 (0.75)	92 (83.6)	Yes	110
8	Cell surface proteomics analysis platforms to define novel and actionable receptors in brain tumours	3.6 (0.57)	58	3.2 (0.71)	32	2.9 (0.72)	16	3.4 (0.68)	94 (88.7)	Yes	106
9	Investigating reasons for treatment resistance	3.5 (0.65)	59	3.5 (0.62)	33	3.6 (0.51)	20	3.5 (0.62)	105 (93.8)	Yes	112
10	Further development of a network for biobanking for all brain tumours	3.6 (0.62)	61	3.3 (0.88)	33	3.3 (0.45)	19	3.4 (0.69)	104 (92.0)	Yes	113
11	Brain tumour registry to track outcomes for all brain tumours	3.6 (0.53)	61	3.1 (0.75)	32	3.5 (0.69)	20	3.4 (0.65)	103 (91.2)	Yes	113
12	Big data repositories/networks for radiation oncology and radiology innovation	3.5 (0.65)	60	3.0 (0.76)	32	3.1 (0.85)	19	3.3 (0.74)	93 (83.8)	Yes	111
13	National benchmarking and quality indicators and outcomes of care (including surgery)	3.6 (0.72)	60	3.2 (0.75)	32	3.4 (0.67)	20	3.4 (0.73)	100 (89.3)	Yes	112
14	Systems for genomic and proteomic profiling of brain tumours	3.6 (0.57)	58	3.4 (0.50)	32	3.4 (0.62)	17	3.5 (0.56)	104 (97.2)	Yes	107
15	Phase 0 studies (imaging, blood, and tumour biomarker development for neoadjuvant therapy)	3.4 (0.63)	57	3.4 (0.55)	32	3.2 (0.95)	17	3.4 (0.67)	97 (91.5)	Yes	106
16	Use of liquid biopsies for diagnosis and monitoring treatment and response	3.5 (0.65)	59	3.2 (0.64)	31	3.1 (0.72)	16	3.3 (0.67)	94 (88.7)	Yes	106
17	Effectiveness of precision medicine/personalised treatment based on genomic profiling	3.6 (0.67)	59	3.4 (0.66)	32	3.6 (0.50)	18	3.5 (0.65)	100 (91.7)	Yes	109
18	Role of theranostics in guiding treatment	3.5 (0.71)	56	3.0 (0.78)	32	3.2 (0.73)	17	3.3 (0.76)	88 (83.8)	Yes	105
19	Clinical trials that include relevant patient-reported outcomes	3.6 (0.58)	59	3.3 (0.79)	32	3.6 (0.69)	19	3.5 (0.67)	101 (91.8)	Yes	110
20	Implementation of research into improving primary care awareness, early diagnosis, and investigation of “red flag” symptoms	3.4 (0.72)	61	2.3 (0.88)	32	3.1 (0.85)	20	3.0 (0.93)	80 (70.8)	No	113
21	Exploring support provided to patients pre-diagnosis and the role of care coordinators pre-diagnosis	3.2 (0.85)	59	2.5 (0.95)	32	3.0 (0.86)	20	2.9 (0.92)	76 (68.5)	No	111
22	Devices/techniques to improve extent of surgical resection	3.3 (0.69)	58	2.8 (0.81)	32	3.1 (0.76)	18	3.2 (0.76)	92 (85.2)	Yes	108
23	Clinical trials using immunotherapy agents	3.6 (0.53)	58	3.3 (0.73)	32	3.3 (0.67)	18	3.5 (0.63)	102 (94.4)	Yes	108
24	Clinical trials using cellular therapies	3.7 (0.47)	56	3.1 (0.78)	32	3.0 (0.65)	15	3.4 (0.68)	94 (91.3)	Yes	103
25	Correlation between chemoresistance and drug metabolism (metabolomics)	3.4 (0.73)	57	3.0 (0.62)	32	2.9 (0.75)	17	3.2 (0.73)	91 (85.8)	Yes	106
26	Determining which drugs are radiation sensitisers and effective in managing brain tumours	3.6 (0.52)	59	2.9 (0.69)	32	3.3 (0.69)	18	3.4 (0.68)	97 (89.0)	Yes	109
27	Drug repurposing studies in adult brain tumours	3.5 (0.63)	58	2.8 (0.87)	32	3.2 (0.75)	16	3.2 (0.78)	87 (82.1)	Yes	106
28	Clinical trials using viral vectors	3.5 (0.61)	54	3.1 (0.78)	32	2.7 (0.80)	15	3.2 (0.74)	83 (82.2)	Yes	101
29	Developing and trialling high throughput in vitro drug screening	3.1 (0.74)	46	2.8 (0.69)	32	2.9 (0.81)	16	2.9 (0.74)	71 (75.5)	Yes	94
30	Trials to address radiation toxicity (acute and late effects)—techniques, survivorship, outcomes	3.5 (0.60)	58	2.8 (0.81)	32	3.3 (0.81)	19	3.3 (0.76)	91 (83.5)	Yes	109
31	Conducting radiation therapy trials to improve outcomes for people with benign brain tumours	3.3 (0.81)	54	2.6 (0.88)	32	3.1 (0.90)	18	3.0 (0.90)	72 (69.2)	No	104
32	Advanced neuro-oncology imaging for diagnosis and treatment response monitoring	3.5 (0.63)	58	3.3 (0.74)	32	3.4 (0.51)	19	3.4 (0.64)	100 (91.7)	Yes	109
33	New effective therapies against rarer primary Central Nervous System tumours	3.4 (0.63)	56	2.9 (0.84)	32	3.2 (0.62)	18	3.2 (0.72)	90 (84.9)	Yes	106
34	Pharmacological/other interventions to improve symptom management	3.4 (0.69)	58	2.8 (0.78)	32	3.2 (0.88)	18	3.2 (0.78)	87 (80.6)	Yes	108
35	Developing treatment utilisation models for standards of care for each main treatment modality	3.3 (0.69)	56	2.6 (0.94)	32	3.2 (0.88)	18	3.1 (0.86)	82 (77.4)	Yes	106
36	Optimal treatment and care pathways for people with brain tumours	3.7 (0.58)	57	3.0 (1.00)	32	3.6 (0.60)	20	3.4 (0.79)	95 (87.2)	Yes	109
37	Identifying barriers to equitable outcomes for under-served populations (e.g., CALD, rural, ATSI) *	3.4 (0.79)	58	2.9 (0.82)	32	3.8 (0.44)	20	3.3 (0.80)	91 (82.7)	Yes	110
38	Determining the impact and optimal models of care coordination	3.3 (0.78)	58	2.8 (0.88)	32	3.3 (0.86)	20	3.2 (0.85)	85 (77.3)	Yes	110
39	Determining the impact and optimal models of telehealth	3.0 (1.00)	59	2.5 (0.84)	32	3.2 (0.67)	20	2.9 (0.93)	73 (65.8)	No	111
40	Determining the impact and developing optimal models of teletrials	3.0 (1.02)	58	2.7 (0.97)	32	3.2 (0.76)	19	2.9 (0.97)	78 (71.6)	No	109
41	Developing and testing interventions for cognitive, personality, and behaviour changes	3.2 (0.83)	59	2.7 (0.82)	32	3.2 (0.83)	20	3.1 (0.85)	80 (72.1)	No	111
42	Developing and testing interventions for fatigue	3.1 (0.88)	59	2.7 (0.85)	32	3.0 (0.97)	20	3.0 (0.89)	77 (69.4)	No	111
43	Effective rehabilitation interventions for patients and carers	3.3 (0.74)	58	2.9 (0.83)	32	3.4 (0.77)	19	3.2 (0.80)	89 (81.7)	Yes	109
44	Exploring the impact of neuro-psychology interventions in brain tumour care	3.2 (0.78)	59	2.6 (0.80)	32	3.1 (0.81)	19	3.0 (0.82)	78 (70.9)	No	110
45	Exploring patients’ and carers’ barriers and enablers in accessing timely palliative care	3.2 (0.86)	57	2.8 (0.84)	32	3.2 (0.89)	20	3.1 (0.88)	81 (74.3)	No	109
46	Exploring and testing palliative care interventions	3.2 (0.85)	57	2.8 (0.76)	32	3.2 (0.89)	20	3.1 (0.85)	81 (74.3)	No	109
47	Evaluating implementation of end-of-life care plans and advance care directives	3.1 (0.98)	56	2.7 (0.82)	32	3.2 (0.88)	20	3.0 (0.93)	76 (70.4)	No	108
48	Evaluating implementation of increased assessment of patient and carer anxiety, distress, and quality of life	3.3 (0.91)	59	2.8 (0.86)	32	3.3 (0.80)	20	3.1 (0.89)	82 (73.9)	No	111
49	Psychosocial interventions for patient/family unmet needs, anxiety, and distress following diagnosis	3.3 (0.86)	58	2.8 (0.78)	32	3.4 (0.68)	20	3.2 (0.83)	84 (76.4)	Yes	110
50	Novel technologies for patients/carers, and their social networks, to support, monitor, and follow-up	3.1 (0.90)	57	2.8 (0.82)	32	3.5 (0.69)	20	3.1 (0.86)	84 (77.1)	Yes	109
51	Exploring grief and loss for patients, carers, and their social networks	3.1 (0.96)	59	2.3 (0.74)	32	3.0 (0.86)	20	2.8 (0.94)	70 (63.1)	No	111
52	Exploring patients’, carers’, and families’ survivorship needs following treatment	3.1 (0.88)	58	2.6 (0.76)	32	3.1 (0.89)	20	2.9 (0.87)	76 (69.1)	No	110
53	Developing and testing survivorship-focused interventions to support patients, carers, and families following treatment	3.1 (0.87)	57	2.6 (0.83)	32	3.3 (0.86)	20	3.0 (0.89)	76 (69.7)	No	109
54	Exploring the financial toxicity associated with brain tumour diagnosis, treatment, and follow-up care	3.1 (0.85)	58	2.3 (0.90)	32	3.1 (0.64)	20	2.9 (0.90)	74 (67.3)	No	110
55	Exploring the cost-effectiveness of supportive care interventions for patients, carers, and their social networks	3.0 (0.97)	58	2.5 (0.80)	32	3.1 (0.64)	20	2.9 (0.90)	73 (66.4)	No	110
56	Developing and testing decision support tools throughout treatment/care pathway to assist patients and carers/families to communicate with clinicians and decide on treatment and supportive care	3.2 (0.96)	59	2.4 (0.80)	32	3.1 (0.91)	20	2.9 (0.96)	75 (67.6)	No	111
57	Trialling interventions to improve patient, carer, and family education about brain tumours, treatment options, disease progression, symptoms, side effects, and supportive care	3.0 (0.91)	59	2.7 (0.75)	32	3.3 (0.73)	20	3.0 (0.86)	79 (71.2)	No	111
58	Determining the role of complementary therapies (e.g., meditation; relaxation; aromatherapy; acupuncture; reflexology; massage) in managing adult brain tumours and how these align with conventional therapies being undertaken	3.1 (0.90)	59	1.8 (0.78)	32	2.4 (0.99)	20	2.6 (1.05)	63 (56.8)	No	111
59	Investigating the role of diet in improving treatment outcomes and managing symptoms and side effects of treatment	3.1 (0.87)	58	1.9 (0.78)	32	2.4 (0.75)	20	2.6 (0.98)	70 (63.6)	No	110
60	Role of exercise: improving treatment outcomes, managing symptoms and treatment side effects	3.3 (0.81)	59	2.6 (0.79)	32	3.0 (0.69)	20	3.0 (0.84)	83 (74.8)	No	111

* CALD—Culturally and Linguistically Diverse, ATSI—Aboriginal and Torres Strait Islander people. The number of responses varied because participants could select ‘unable to score’ (at least one participant (range of *n* = 1–17) selected this in 44 priorities) or could skip answering priorities. There is a slight attrition towards the end of the list (ten priorities presented per page) due to some participants (*n* = 4 consumers, *n* = 1 health professional) dropping out of the survey.

**Table 5:** Phase 2, Step 2—Participants’ selection of the top ten research priorities and adjusted mean rankings (higher mean equals higher priority).

Rank Posit-ion	Research Priority	Refer-ence Number in Step 1	Top Ten Selections by Participants *n* = 89		Ranking			
			Selected	Not Selected	Consumers *n* = 42	Health Professionals *n* = 23	Researchers *n* = 17	Average Mean *n* = 82
			*n* (%)	*n* (%)	Mean (SD)	Mean (SD)	Mean (SD)	Mean (SD)
1	Clinical trials that include relevant patient-reported outcomes	19	38 (42.7)	51 (57.3)	**3.5 (4.25)** ^a, b^	1.3 (2.60) ^b^	**4.8 (4.32)** ^a^	3.2 (3.72) *
2	Understanding of tumour microenvironment and immunosuppression in immunotherapy	5	43 (48.3)	46 (51.7)	**3.1 (3.78)**	**3.3 (3.63)**	**2.7 (4.12)**	**3.0 (3.84)**
3	Effectiveness of precision medicine/personalised treatment based on genomic profiling	17	42 (47.2)	47 (52.8)	**2.9 (3.72)**	**3.3 (3.58)**	**2.2 (3.44)**	**2.8 (3.58)**
4	Pre-clinical research to identify actionable drivers (and new molecular targets) for therapy	3	33 (37.1)	56 (62.9)	**2.0 (3.39)** ^b^	**4.3 (4.09)** ^a^	1.2 (2.46) ^b^	2.5 (3.31) *
5	Advanced neuro-oncology imaging for diagnosis and treatment response monitoring	32	37 (41.6)	52 (58.4)	1.9 (3.17)	**3.0 (3.13)**	**2.2 (2.84)**	**2.4 (3.05)**
6	Investigating reasons for treatment resistance	9	31 (34.8)	58 (65.2)	**2.0 (3.41)**	**2.5 (3.68)**	**2.1 (3.28)**	**2.2 (3.46)**
7	Brain tumour registry to track outcomes for all brain tumours	11	40 (44.9)	49 (55.1)	**2.9 (3.57)**	**2.3 (4.05)**	1.5 (2.65)	**2.2 (3.42)**
8	Pre-clinical models and strategies to enhance Blood Brain Barrier (BBB) penetration for novel drugs	6	34 (38.2)	55 (61.8)	**2.7 (3.32)**	2.0 (3.05)	1.8 (2.99)	**2.2 (3.12)**
9	National benchmarking and quality indicators and outcomes of care (including surgery)	13	29 (32.6)	60 (67.4)	1.3 (2.65)	**2.5 (3.50)**	**2.3 (3.69)**	**2.0 (3.28)**
10	Clinical trials using immunotherapy agents	23	31 (34.8)	58 (65.2)	**2.7 (3.87)**	1.3 (2.72)	1.8 (3.36)	**1.9 (3.32)**
11	Phase 0 studies (imaging, blood, and tumour biomarker development for neoadjuvant therapy)	15	24 (27.0)	65 (73.0)	0.8 (1.96) ^b^	**4.1 (4.38)** ^a^	0.7 (2.20) ^b^	1.9 (2.85) **
12	Understanding the causes of adult brain tumour development	1	32 (36.0)	57 (64.0)	**3.6 (3.96)** ^a^	0.0 (0.00) ^b^	1.8 (3.17) ^a, b^	1.8 (2.38) **
13	Use of liquid biopsies for diagnosis and monitoring treatment and response	16	28 (31.5)	61 (68.5)	1.5 (2.95) ^b^	**3.0 (3.21)** ^a^	0.8 (1.79) ^b^	1.8 (2.65) *
14	Psychosocial interventions patient/family unmet needs, anxiety, and distress following diagnosis	49	28 (31.5)	61 (68.5)	1.3 (2.24) ^a, b^	0.3 (0.93) ^b^	**3.6 (4.53)** ^a^	1.8 (2.57) *
15	Optimal treatment and care pathways for people with brain tumours	36	25 (28.1)	64 (71.9)	1.5 (2.82)	0.6 (2.21)	**2.7 (3.67)**	1.6 (2.90)
16	Improved pre-clinical models of gliomas and other primary Central Nervous System malignancies	4	22 (24.7)	67 (75.3)	1.1 (2.55)	2.2 (3.41)	1.4 (2.29)	1.5 (2.75)
17	Further development of a network for biobanking for all brain tumours	10	28 (31.5)	61 (68.5)	1.9 (3.06)	**2.3 (3.51)**	0.4 (1.06)	1.5 (2.54)
18	Clinical trials using cellular therapies	24	20 (22.5)	69 (77.5)	**2.2 (3.59)**	1.2 (2.48)	1.1 (3.01)	1.5 (3.03)
19	Systems for genomic and proteomic profiling of brain tumours	14	17 (19.1)	72 (80.9)	0.7 (1.94)	1.5 (2.81)	1.8 (3.36)	1.3 (2.70)
20	Identifying barriers to equitable outcomes for under-served populations (e.g., CALD, rural, ATSI)	37	19 (21.3)	70 (78.7)	1.0 (2.44)	1.0 (2.57)	**1.9 (2.64)**	1.3 (2.55)
21	Determining the impact and optimal models of care coordination	38	16 (18.0)	73 (82.0)	0.7 (2.13) ^b^	0.3 (1.25) ^b^	**2.6 (3.77)** ^a^	1.2 (2.38)**
22	Determining which drugs are radiation sensitisers and effective in managing brain tumours	26	24 (27.0)	65 (73.0)	1.3 (2.59)	0.6 (1.44)	1.6 (2.55)	1.2 (2.19)
23	New effective therapies against rarer primary Central Nervous System tumours	33	19 (21.3)	70 (78.7)	1.0 (2.74)	1.2 (2.29)	1.2 (2.70)	1.2 (2.58)
24	Trials to address radiation toxicity (acute and late effects)—techniques, survivorship, outcomes	30	25 (28.1)	64 (71.9)	1.0 (2.06)	1.1 (2.68)	1.2 (1.95)	1.1 (2.23)
25	Drug repurposing studies in adult brain tumours	27	21 (23.6)	68 (76.4)	**2.0 (3.04)** ^a^	0.4 (1.34) ^b^	0.5 (2.18) ^b^	1.0 (2.19) **
26	Cell surface proteomics analysis platforms to define novel and actionable receptors in brain tumours	8	18 (20.2)	71 (79.8)	0.8 (1.68)	1.7 (2.96)	0.5 (1.74)	1.0 (2.13)
27	Novel therapeutic approaches with the ability to specifically target the stem cell-like population	7	20 (22.5)	69 (77.5)	1.2 (2.43)	1.0 (1.89)	0.7 (2.11)	1.0 (2.14)
28	Effective rehabilitation interventions for patients and carers	43	15 (16.9)	74 (83.1)	1.0 (2.51)	0.2 (0.65)	1.5 (2.76)	0.9 (1.97)
29	Role of theranostics in guiding treatment	18	16 (18.0)	73 (82.0)	0.1 (0.48) ^b^	1.8 (3.35) ^a^	0.7 (1.69) ^a, b^	0.9 (1.84) **
30	Clinical trials using viral vectors	28	15 (16.9)	74 (83.1)	1.0 (2.37)	1.1 (2.58)	0.3 (1.21)	0.8 (2.05)
31	Devices/techniques to improve the extent of surgical resection	22	18 (20.2)	71 (79.8)	1.0 (2.19)	1.0 (2.43)	0.3 (0.99)	0.7 (1.87)
32	Novel technologies for patients/carers, and their social networks, to support, monitor, and follow-up	50	14 (15.7)	75 (84.3)	0.4 (1.03)	0.2 (0.65)	1.6 (3.04)	0.7 (1.57)
33	Big data repositories/networks for radiation oncology and radiology innovation	12	11 (12.4)	78 (87.6)	0.5 (1.77)	0.8 (2.26)	0.8 (2.11)	0.7 (2.05)
34	Role of exercise: improving treatment outcomes, managing symptoms and treatment side effects	60	19 (21.3)	70 (78.7)	1.0 (2.28)	0.2 (0.74)	0.8 (1.63)	0.7 (1.55)
35	Pharmacological/other interventions to improve symptom management	34	15 (16.9)	74 (83.1)	0.6 (1.56)	0.3 (1.26)	0.8 (2.33)	0.6 (1.72)
36	Developing treatment utilisation models for standards of care for each main treatment modality	35	10 (11.2)	79 (88.8)	0.1 (0.37)	0.4 (1.88)	1.1 (2.71)	0.5 (1.65)
37	Correlation between chemoresistance and drug metabolism (metabolomics)	25	13 (14.6)	76 (85.4)	0.6 (1.94)	0.7 (1.75)	0.0 (0.00)	0.4 (1.23)

**Bold**—Research priority mean ranking in the top ten for means. * 0.01 ≤ *p* < 0.05, ** *p* < 0.01. ^a, b^ Different notation shows group means which are significantly different in post-hoc pair-wise comparisons (Bonferroni adjusted significance).

## Data Availability

The data presented in this study are available on request from the corresponding author.

## Supplementary Materials

The following supporting information can be downloaded at: https://www.mdpi.com/article/10.3390/curroncol29120781/s1, File S1: Phase 2, Step 1 online survey instrument; Figure S1: Radar figure of each sub-group’s ranking of the overall top fifteen priorities; Figure S2: Line graph figure of the change in overall and sub-group mean rankings of the 1st to 37th ranked priority shown in Table 5.
