# Supplementary material for: Determining the Research Priorities for Adult Primary Brain Tumours in Australia and New Zealand: A Delphi Study with Consumers, Health Professionals, and Researchers
Source: Curr Oncol. 2022 Dec 17;29(12):9928–55. doi: 10.3390/curroncol29120781 (PMC9777470; doi:10.3390/curroncol29120781)
Supplement: Supplementary file 1 [file curroncol-29-00781-s001.zip › curroncol-2054389-supplementary.pdf]

Supplementary Materials File S1

Phase 2 - Brain Tumor Delphi

Participant information and eligibility questions

Please provide your contact details so we can contact you about the future rounds of the study.

- ○ Preferred name ________________________________
- ○ Email ________________________________

What is currently your main role as it relates to adults with primary brain tumours?

- ○ Health professional
- ○ Researcher
- ○ Consumer (patient/carer/advocate)

In which country do you currently live?

- ○ Australia
- ○ New Zealand
- ○ Neither

End of Block: Participant information and eligibility questions

---

Start of Block: Consumer demographics

Please indicate your main background experience with adult primary brain tumours

- ○ Previously diagnosed with a primary brain tumour
- ○ Carer for patient with a primary brain tumour
- ○ Advocate
- ○ Other, please specify ________________________________

What is your postcode?

____

Please indicate where you live?

- ○ Major city
- ○ Regional/Rural
- ○ Remote

Please tell us about your education background?

- ○ Did not complete high school
- ○ Completed high school
- ○ TAFE certificate/ diploma, Business College
- ○ University degree
- ○ Higher degree (postgraduate)

What is your age?

	18	25	32	40	47	54	61	68	76	83	90
Years											

What is your gender?

- ○ Male
- ○ Female
- ○ Other (please state) ____
- ○ Prefer not to answer

Display This Question:

If Please indicate your main background experience with adult primary brain tumours = Previously diagnosed with a primary brain tumour

The following questions are about the treatment you have received for a brain tumour.

Display This Question:

If Please indicate your main background experience with adult primary brain tumours = Carer for patient with a primary brain tumour

Q84 The following questions are about or the treatment your relative or friend has received for an adult primary brain tumour.

Display This Question:

If Please indicate your main background experience with adult primary brain tumours = Previously diagnosed with a primary brain tumour

Or Please indicate your main background experience with adult primary brain tumours = Carer for patient with a primary brain tumour

Q15 Please indicate the time since you, or your relative or friend, completed treatment?

	0 2 4 6 8 10 12 14 16 18 20
Years since treatment completed	

Display This Question:

If Please indicate your main background experience with adult primary brain tumours = Previously diagnosed with a primary brain tumour

Or Please indicate your main background experience with adult primary brain tumours = Carer for patient with a primary brain tumour

Q16 In which location did you, or your relative or friend, receive treatment?

- ○ Major city
- ○ Regional/Rural
- ○ Remote

Display This Question:

If Please indicate your main background experience with adult primary brain tumours = Previously diagnosed with a primary brain tumour

Or Please indicate your main background experience with adult primary brain tumours = Carer for patient with a primary brain tumour

Q17 Please indicate if you, or your relative or friend, received treatment in the following settings:

- ○ Hospital - Public
- ○ Hospital - Private
- ○ Private - Practice
- ○ Both Public and Private
- ○ Other (please specify) ____
- ○ Unsure

End of Block: Consumer demographics

Start of Block: Health professional demographics

Q18 What is the postcode of your primary workplace?

____

Q19 Please indicate the nature of your primary workplace location?

- ○ Major city
- ○ Regional/Rural
- ○ Remote

Q20 Is your main interaction with patients diagnosed with adult primary brain tumours in...?

- ○ Hospital - Public
- ○ Hospital - Private
- ○ Private - Practice
- ○ Both Public and Private
- ○ Other, please specify ____

Q21 Type of clinical setting

- ○ Tertiary referral cancer center
- ○ District/local hospital
- ○ Non-hospital based practice
- ○ Other, please specify ____
- ○ N/A

Q22 On average, approximately how many adult patients with a new diagnosis of primary brain tumour, does your primary site of practice see per year?

	0 20 40 60 80 100 120 140 160 180 200
Average number of new patients	

Q23 Which of the following best describes your role in relation to the management of adults with primary brain tumours?

- ◯ Medical oncologist
- ◯ Neuro-oncologist
- ◯ Radiation oncologist
- ◯ Neurosurgeon
- ◯ Nurse
- ◯ Cancer Care Coordinator
- ◯ Physiotherapist
- ◯ Occupational therapist
- ◯ Social Worker
- ◯ Psychiatrist
- ◯ Clinical psychologist or psycho-oncologist
- ◯ Palliative care physician
- ◯ Rehabilitation physician
- ◯ Trainee (please specify discipline) ____
- ◯ Other, please specify ____

Q24 Years practising in your discipline?

	0	4	8	12	16	20	24	28	32	36	40
Number of years											

Q25 Years practising in your current position?

	0	4	8	12	16	20	24	28	32	36	40
Number of years											

Q26 What is your highest level of research training?

- ○ Completed a Master of Research (or equivalent)
- ○ Currently enrolled in a Master of Research (or equivalent)
- ○ Completed a Doctor of Philosophy (or equivalent)
- ○ Currently enrolled in a Doctor of Philosophy (or equivalent)
- ○ None of the above

Q27 What experience do you have in relation to adult primary brain tumour research?

- ○ No experience
- ○ Predominantly referral of patients to clinical trials only
- ○ Minimal experience (e.g., involvement in a research project)
- ○ Some experience (e.g., trial co-investigator)
- ○ Substantial experience (e.g., being the chief or principal investigator on a primary brain tumour research study)
- ○ Research assistant/research officer
- ○ Other, please specify ____

Q28 What is your age?

	18	25	32	40	47	54	61	68	76	83	90
Age in years											

Q29 What is your gender

- ○ Male
- ○ Female
- ○ Other (please state) ____
- ○ Prefer not to answer

End of Block: Health professional demographics

Start of Block: Researchers or others

Q30 What is the postcode of your primary workplace

____

Q31 Please indicate the nature of your primary workplace location?

- ○ Major city
- ○ Regional/Rural
- ○ Remote

Q32 Please indicate the research setting you work in?

- ○ University
- ○ Hospital
- ○ Both (University and Hospital)
- ○ Other (please specify) ____

Q33 Please describe the type of research you undertake in relation to adult primary brain tumour research.

____
____
____
____
____

Q34 Years practising in your discipline?

	0	4	8	12	16	20	24	28	32	36	40
Years in discipline											

Q35 Years practising in your current position?

	0	4	8	12	16	20	24	28	32	36	40
Years in current position											

Q36 What is your highest level of research training?

- ○ Completed a Master of Research (or equivalent)
- ○ Currently enrolled in a Master of Research (or equivalent)
- ○ Completed a Doctor of Philosophy (or equivalent)
- ○ Currently enrolled in a Doctor of Philosophy (or equivalent)
- ○ None of the above

Q37 What experience do you have in relation to adult primary brain tumour research?

- ○ No experience
- ○ Minimal experience (e.g., involvement in a primary brain tumour research project)
- ○ Some experience (e.g., trial co-investigator)
- ○ Substantial experience (e.g., being the chief or principal investigator on a primary brain tumour research study)
- ○ Research assistant/research officer
- ○ Other (please specify) ____

Q38 What is your age?

	18 25 32 40 47 54 61 68 76 83 90
Age in years	

Q39 What is your gender?

- ○ Male
- ○ Female
- ○ Other (please state) ____
- ○ Prefer not to answer

End of Block: Researchers or others

Start of Block: Survey questions - all participants

Q40 The aim of the study is to determine the priorities for adult primary brain tumour research in Australia and New Zealand. Adult primary brain tumours include primary malignant or benign brain tumours diagnosed in people aged 18 years or older. The priorities that have been identified in the list provided focus on research and clinical trials rather than the quality of clinical care per se.

On the next pages you will see a list of research priorities that were generated from Phase 1. Please rate each priority based on the importance you feel it holds for research on adult brain tumours in Australia and New Zealand, on the scale provided.

1. not important; not a priority
2. somewhat important; low priority
3. moderately important; intermediate priority
4. very important; urgent priority

Items may be rated as “very important” if they address one or more of the following aspects:

1. Research is urgently needed in the area.
2. There is a gap in the existing evidence base in that area
3. Research in the area will directly impact the quality of life of patients diagnosed with a primary brain tumour and their carers/families

We have provided definitions for some of the scientific terms and concepts directly beneath each priority where deemed relevant.

Should the meaning still be unclear, we have provided an “unable to score” option, but we ask that you use this sparingly.

Please indicate how important each research priority is to you.

	1 Not important; not a priority	2 Somewhat important; low priority	3 Moderately important; intermediate priority	4 Very important; urgent priority	X Unable to score
**1. Understanding the causes of adult brain tumour development.**	○	○	○	○	○
**2. Developing research questions around familial glioma syndromes.**	○	○	○	○	○
**3. Conducting pre-clinical research to identify actionable drivers (and new molecular targets) for brain tumour therapy.** *Conducting laboratory research in animals (pre-clinical) to identify genetic changes in brain tumours that will justify use of targeted therapy in patients.*	○	○	○	○	○
**4. Development of pre-clinical models of gliomas and other primary Central Nervous System malignancies with improved validity and reproducibility.** *Developing reliable and accurate ways of predicting personalised responses to new drugs in animals (pre-clinical), taking into account personalised tumour characteristics.*	○	○	○	○	○
**5. Understanding the tumour microenvironment with the aim of reducing immunosuppression and facilitating immunotherapy.** *Understanding the interaction between tumours and their surrounding tissue (tumour microenvironment) to reduce immunosuppression and facilitate immunotherapy.*	○	○	○	○	○
**6. Developing pre-clinical models and strategies to enhance Blood Brain Barrier (BBB) penetration for novel drugs.** *Developing methods to increase penetration of new drugs into the brain.*	○	○	○	○	○
**7. Developing and testing of novel therapeutic approaches with the ability to specifically target the stem cell-like**	○	○	○	○	○

population. *(Stem cells are special human cells that are able to develop into many different cell types).*

**8. Developing cell surface proteomics analysis platforms to better define novel and actionable receptors in brain tumours.** *Developing sophisticated ways of analysing proteins expressed on the surface of tumours (proteomics) to allow us to specifically target them.*	○	○	○	○	○
**9. Investigating reasons for treatment resistance.**	○	○	○	○	○
**10. Further development of a network for biobanking for all brain tumours.** *Development of infrastructure to enable storage of brain tumour samples (biobanking) for future research.*	○	○	○	○	○

Please provide any comments related to your answers for the items above (1-10).
Comments might include why you rated items as high priority or not a priority. You may also clarify why you have selected "unable to score" for any items.

____

Please indicate how important each research priority is to you.

	1 Not important; not a priority	2 Somewhat important; low priority	3 Moderately important; intermediate priority	4 Very important; urgent priority	X Unable to score
**11. Developing a brain tumour registry to track outcomes for all brain tumours in Australia and New Zealand**.	○	○	○	○	○
**12. Developing big data repositories/networks for radiation oncology and radiology innovation.**	○	○	○	○	○
**13. Developing national benchmarking and quality indicators and outcomes of care (including surgery) to improve the quality and efficacy of treatment.**	○	○	○	○	○
**14. Developing systems for genomic and proteomic profiling of brain tumours.**	○	○	○	○	○
**15. Conducting Phase 0 studies (including imaging, blood and tumour biomarker development to enable neoadjuvant therapy).** *Conducting drug dosing studies to show drug penetration into the brain (with or without treatment benefit) and then developing ways of measuring treatment response by testing blood or tumour tissue when drugs are given prior to surgery (Phase 0).*	○	○	○	○	○
**16. Evaluating the use of liquid biopsies for diagnosis and monitoring treatment and response.** *(A liquid biopsy is a test done on a sample of blood to look for cancer cells that are circulating in the blood).*	○	○	○	○	○
**17. Exploring the effectiveness of precision medicine/personalised treatment based on genomic profiling.** *Exploring the effectiveness of personalising cancer treatment based on an individual's tumour mutations.*	○	○	○	○	○

**18. Developing the role of theranostics in guiding treatment.** *Developing the role of using a personalised approach which can be used as both a) diagnostic and b) therapy tools (theranostics).*	○	○	○	○	○
**19. Conducting clinical trials that include relevant patient reported outcomes** (including Quality of Life or unmet supportive care needs).	○	○	○	○	○
**20. Implementation research into improving primary care awareness, early diagnosis and investigation of "red flag" symptoms** (implementation of optimal care pathway and demonstrating impact of delay).	○	○	○	○	○

Q44 **Please provide any comments related to your answers for the items above (11-20).** *Comments might include why you rated items as high priority or not a priority. You may also clarify why you have selected "unable to score" for any items.*

____

Please indicate how important each research priority is to you.

	1 Not important; not a priority	2 Somewhat important; low priority	3 Moderately important; intermediate priority	4 Very important; urgent priority	X Unable to score
**21. Exploring support provided to patients pre-diagnosis and the role of care coordinators pre-diagnosis.**	○	○	○	○	○
**22. Testing devices/techniques to improve extent of surgical resection** (e.g. focused ultrasonography)**.**	○	○	○	○	○
**23. Development of clinical trials using immunotherapy agents.**	○	○	○	○	○
**24. Conducting clinical trials using cellular therapies** (e.g. CAR T cells, dendritic cells)**.**	○	○	○	○	○
**25. Determining the correlation between chemoresistance and drug metabolism (metabolomics).** *Determining the relationship between tumour resistance (chemoresistance) to chemotherapy/drug therapy and drug metabolism (metabolomics).*	○	○	○	○	○
**26. Determining which drugs are radiation sensitisers that are effective in managing brain tumours.** *Determining which drugs can be used to enhance the effectiveness of radiation (radiation sensitisers) used to treat brain tumours.*	○	○	○	○	○
**27. Conducting drug repurposing studies in adult brain tumours.** *Conducting studies of drugs useful for other conditions to test their effectiveness in brain tumours.*	○	○	○	○	○
**28. Conducting clinical trials using viral vectors** (e.g. oncolytic viruses, viral gene therapy)**.**	○	○	○	○	○
**29. Developing and trialling high throughput in vitro drug screening**	○	○	○	○	○

**30. Developing trials to address mitigation of radiation toxicity (acute and late radiation effects) – new techniques, survivorship, improved outcomes.**	○	○	○	○	○

Q46 **Please provide any comments related to your answers for the items above (21-30).**
Comments might include why you rated items as high priority or not a priority. You may also clarify why you have selected "unable to score" for any items.

____

Q47 Please indicate how important each research priority is to you.

	1 Not important; not a priority	2 Somewhat important; low priority	3 Moderately important; intermediate priority	4 Very important; urgent priority	X Unable to score
**31. Conducting radiation therapy trials to improve outcomes for people with benign brain tumours** (e.g. meningiomas)**.**	○	○	○	○	○
**32. Investigating advanced neuro-oncology imaging (e.g. newer MRI and PET scanning techniques) for diagnosis and treatment response monitoring.**	○	○	○	○	○
**33. Developing new effective therapies against rarer primary Central Nervous System tumours.**	○	○	○	○	○
**34. Developing and testing of pharmacological/other interventions to improve symptom management** (including seizures, thromboembolic events (blood clotting events))**.**	○	○	○	○	○
**35. Developing actual versus optimal utilisation models for standards of care** (for each main treatment modality e.g. surgery/radiotherapy/systemic therapies)**.**	○	○	○	○	○
**36. Evaluating optimal treatment and care pathways for people with brain tumours.**	○	○	○	○	○
**37. Identifying barriers to equitable outcomes for under-served populations** (including regional/remote patients, culturally and linguistically diverse populations, and First Nations/ Aboriginal and Torres Strait Islander/Māori and Pasifika people)**.**	○	○	○	○	○
**38. Determining the impact and optimal models of care coordination.**	○	○	○	○	○

**39. Determining the impact and optimal models of telehealth** (to allow patients to participate in healthcare appointments virtually).	○	○	○	○	○
**40. Determining the impact and developing optimal models of teletrials** (to allow participation and conduct of clinical trials virtually).	○	○	○	○	○

Q48 **Please provide any comments related to your answers for the items above (31-40).**
Comments might include why you rated items as high priority or not a priority. You may also clarify why you have selected "unable to score" for any items.

____

Q49 Please indicate how important each research priority is to you.

	1 Not important; not a priority	2 Somewhat important; low priority	3 Moderately important; intermediate priority	4 Very important; urgent priority	X Unable to score
**41. Developing and testing interventions for cognitive, personality and behaviour changes.**	○	○	○	○	○
**42. Developing and testing interventions for fatigue.**	○	○	○	○	○
**43. Determining the most effective rehabilitation interventions for patients and carers.**	○	○	○	○	○
**44. Exploring the impact of neuro-psychology interventions in brain tumour care.**	○	○	○	○	○
**45. Exploring patients and carers' barriers and enablers in accessing timely palliative care.**	○	○	○	○	○
**46. Exploring and testing palliative care interventions.**	○	○	○	○	○
**47. Evaluating implementation of end-of-life care plans and advance care directives.**	○	○	○	○	○
**48. Evaluating implementation of increased assessment of patient and carer anxiety, distress, and quality of life.**	○	○	○	○	○
**49. Developing and testing psychosocial interventions to address unmet needs, anxiety, and distress experienced by patients, carers, and families following a brain tumour diagnosis.**	○	○	○	○	○
**50. Trialling novel technologies for delivering support, monitoring, and follow-up to patients, carers, and their social networks.**	○	○	○	○	○

Q50 **Please provide any comments related to your answers for the items above (41-50).**
Comments might include why you rated items as high priority or not a priority. You may also clarify why you have selected "unable to score" for any items.

____

Q51 Please indicate how important each research priority is to you.

	1 Not important; not a priority	2 Somewhat important; low priority	3 Moderately important; intermediate priority	4 Very important; urgent priority	X Unable to score
**51. Exploring grief and loss (e.g. loss of independence, loss of the person who once was, loss of the imagined future) for patients, carers and their social networks.**	○	○	○	○	○
**52. Exploring patients, carers and families survivorship needs following treatment.**	○	○	○	○	○
**53. Developing and testing survivorship focused interventions to support patients, carers and families following treatment.**	○	○	○	○	○
**54. Exploring the financial toxicity associated with brain tumour diagnosis, treatment, and follow up care.**	○	○	○	○	○
**55. Exploring the cost-effectiveness of supportive care interventions for patients, carers and their social networks.**	○	○	○	○	○
**56. Developing and testing decision support tools throughout treatment/care pathway to assist patients and carers/families to communicate with clinicians and decide on treatment and supportive care.**	○	○	○	○	○
**57. Trialling interventions to improve patient, carer and family education about brain tumours, treatment options, disease progression, symptoms and side effects and supportive care.**	○	○	○	○	○

**58. Determining the role of complementary therapies (e.g. meditation; relaxation; aromatherapy; acupuncture; reflexology; massage) in managing adult brain tumours and how these align with conventional therapies being undertaken.**	○	○	○	○	○
**59. Investigating the role of diet in improving treatment outcomes and managing symptoms and side effects of treatment.**	○	○	○	○	○
**60. Investigating the role of exercise in improving treatment outcomes and managing symptoms and side effects of treatment.**	○	○	○	○	○

Q52 **Please provide any comments related to your answers for the items above (51-60).**
Comments might include why you rated items as high priority or not a priority. You may also clarify why you have selected "unable to score" for any items.

____

Q53 Please outline any research priorities for adult brain tumours in Australia and New Zealand that you believe are missing or not adequately covered in this survey:

____

Q54 Please leave any further comments that you feel may be useful to determining research priorities for adult brain tumours in Australia and New Zealand:

____

End of Block: Survey questions - all participants

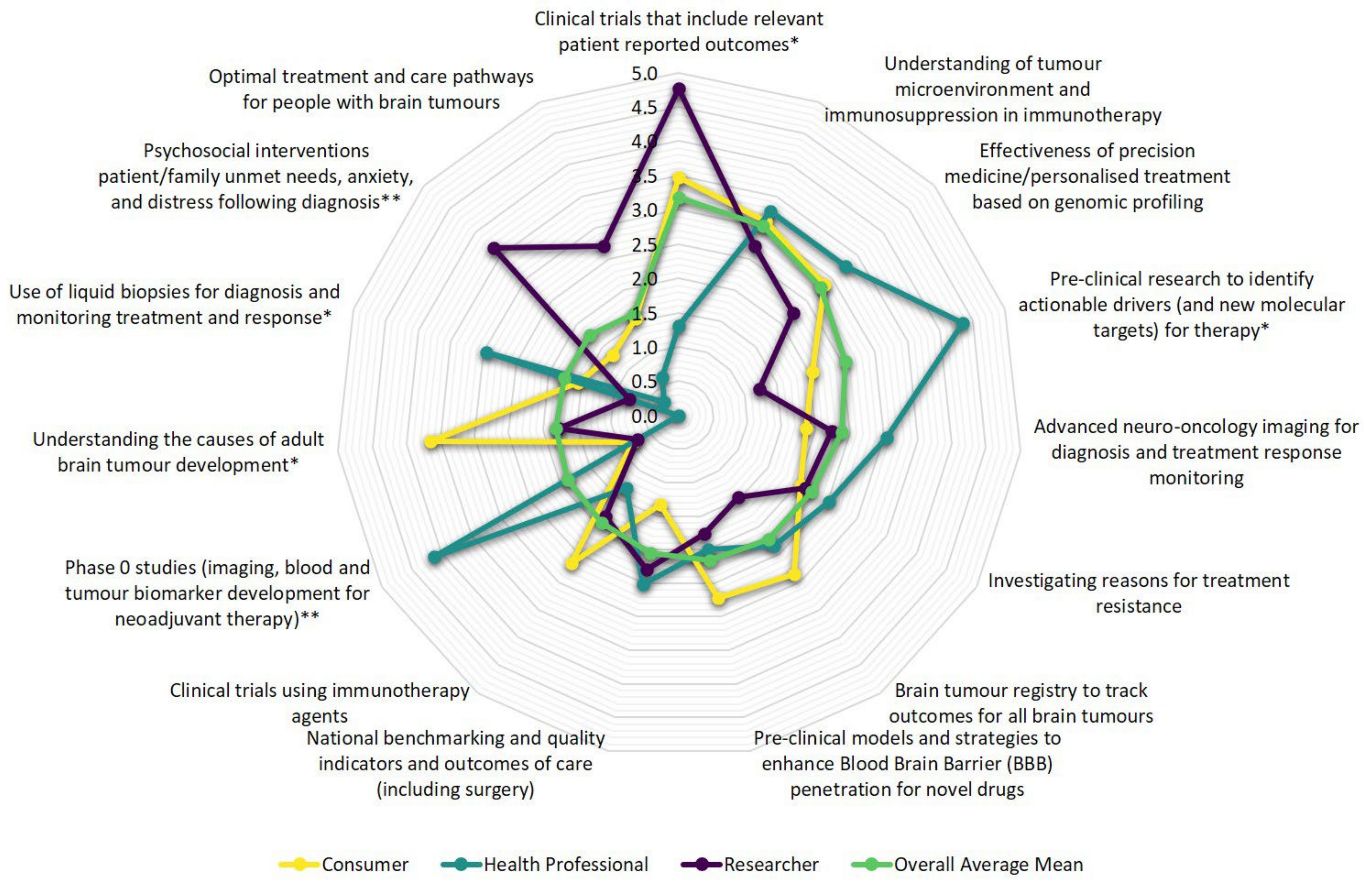

Figure S1. Mean adjusted rankings for top fifteen ranked research priorities by group and overall average mean (higher mean equals higher priority). Priorities are shown from highest to lowest overall mean (from top in clockwise direction). Priorities with notation (*$0.01 \leq p < 0.05$, **$p < 0.01$) have significant inter-group differences.

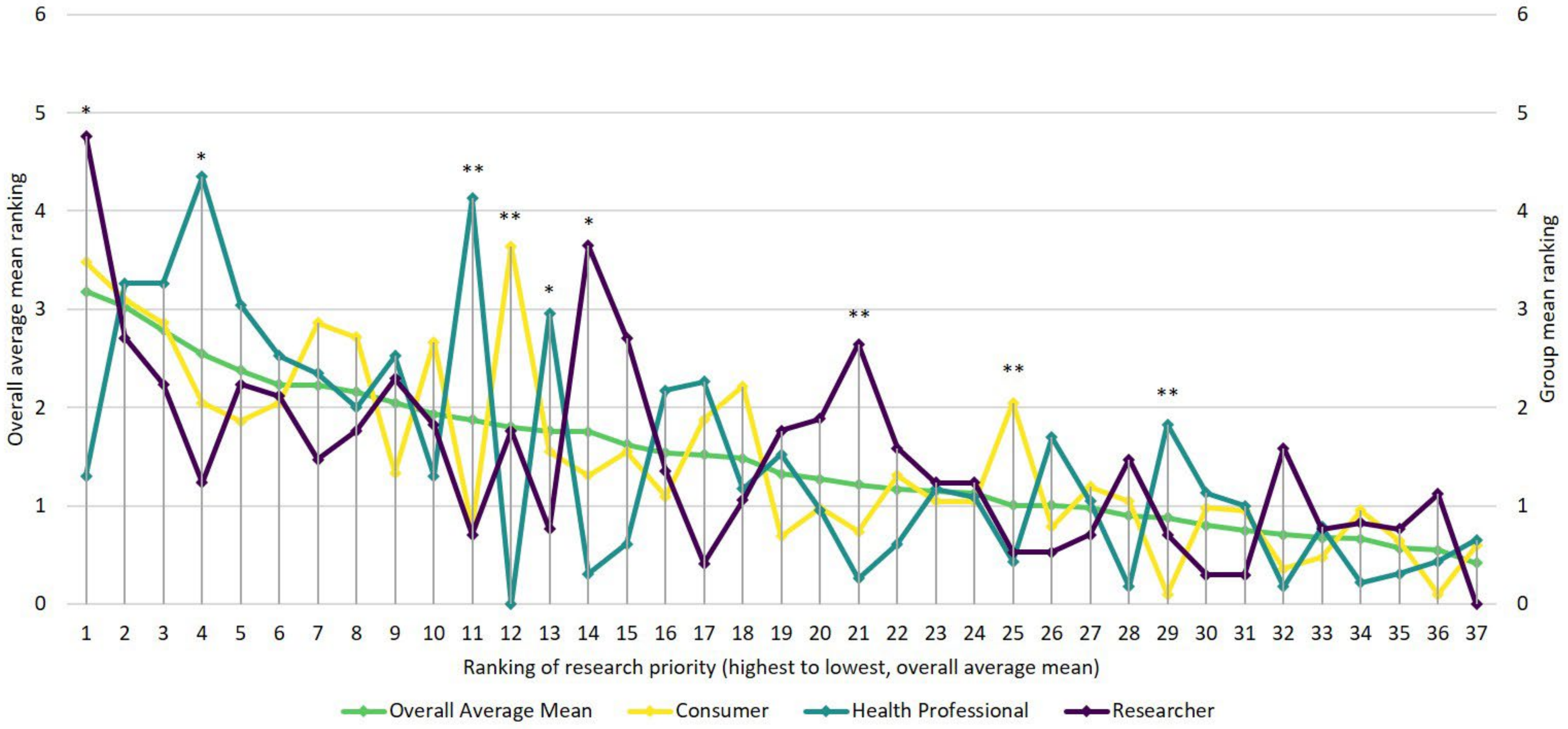

Figure S2. Mean adjusted rankings for all 37 research priorities in phase 2, step 2 by overall average mean (left axis) and by group mean (right axis). Higher mean equals higher priority. Refer to rank position column in table 5 for wording of each priority. Priorities with notation (*$0.01 \le p < 0.05$, **$p < 0.01$) have significant inter-group differences.
